# *Jasminum parceflorum* (Oleaceae), a new species from southern Yunnan, China

**DOI:** 10.3897/phytokeys.146.49625

**Published:** 2020-05-08

**Authors:** Kai Zhang, Mingsong Wu, Bo Pan, Lianxuan Zhou, Dianxiang Zhang

**Affiliations:** 1 Key Laboratory of Plant Resources Conservation and Sustainable Utilization, South China Botanical Garden, Chinese Academy of Sciences, Guangzhou 510650, Guangdong, China South China Botanical Garden, Chinese Academy of Sciences Guangdong China; 2 Centre for Integrative Conservation, Xishuangbanna Tropical Botanical Garden, Chinese Academy of Sciences, Mengla 666303, Yunnan, China University of Chinese Academy of Sciences Beijing China; 3 University of Chinese Academy of Sciences, Beijing 100049, China Xishuangbanna Tropical Botanical Garden, Chinese Academy of Sciences Mengla China

**Keywords:** *
Jasminum
*, limestone forest, Xishuangbanna

## Abstract

*Jasminum parceflorum* (Oleaceae), a new species from tropical limestone habitats in Yunnan, China, is described and illustrated here. The new species is similar to *J. pierreanum* and *J. rarum*, but can be distinguished by its linear calyx lobes, dry calyces without ridges, terminal 1 (or 3)-flowered cymes and axillary solitary flowers.

## Introduction

*Jasminum* L. (Linnaeus 1753), with about 200 species of woody climbers and erect shrubs (Green 2004), is the largest genus of the olive family. The genus is distributed throughout the Old World tropics and warm temperate regions (Green 1969, 2004), with the specific diversity center suited in tropical Asia. Within *Jasminum*, De Candolle (1844) divided the genus into four sections based on leaf arrangement and leaf organization, i.e., sect. Unifoliolata DC., sect. Trifoliolata DC., sect. Alternifolia DC., and sect. Jasminum (sect. Pinnatifolia DC.). Green (2001) followed De Candolle’s infrageneric classification and further added another section, i.e., sect. Primulina P. S. Green, which is characterized by the combination of opposite leaves and yellow flowers. In China, more than 40 species of *Jasminum* have been recorded (Chang et al. 1996, Zhang et al. 2019), of which at least 26 species can be found in limestone forests.

During a botanical expedition to Yinchang Mountain, Jinghong City, Yunnan Province, China in February 2018, we encountered an unusual species lacking flower and fruit, which possesses the most typical character states of the genus *Jasminum* such as opposite leaves, entire leaf margin, articulated petiole, abaxial vein axils with tufted hairs and abaxial leaf surface with glandular dots. However, based on some other vegetative characters such as laminar size, shape and vein characters, etc., the species was distinctly different from any described jasmine species in China and neighboring countries. In order to know more about its habitat and characters of its flowering and fruiting, we revisited Yinchang Mountain and adjacent areas in July 2018, July and November 2019, and collected more specimens. Subsequent detailed morphological comparisons with the similar species revealed that it represents an undescribed *Jasminum* species, belonging to J. sect. Unifoliolata DC. (De Candolle 1844) by having simple, opposite leaves and white flowers.

## Materials and methods

Observations and measurements of morphological characters of the new species were carried out in the field and at the herbarium, based on living individuals and specimens. Glandular dots, hairs and other tiny morphological characters were observed by using a stereomicroscope (LEICA EZ4W). Morphological comparisons with related species based on the specimens from the JSTOR Global Plants (http://plants.jstor.org/), IBK, IBSC, KUN and SYS herbaria.

## Taxonomy

### Jasminum parceflorum

Taxon classificationPlantaeLamialesOleaceae

Kai Zhang & D.X. Zhang sp. nov.

1D6A07D3-49EF-57B4-ACC5-194D119271C4

urn:lsid:ipni.org:names:77209570-1

Figs 1 , 2

#### Diagnosis.

*Jasminum parceflorum* is morphologically similar to *J. pierreanum* Gagnep. and *J. rarum* Kerr, but can be distinguished by its linear calyx lobes, dry calyces without ridges, terminal 1 (or 3)-flowered cymes and axillary solitary flowers.

#### Type.

CHINA. Yunnan Province: Xishuangbanna Prefecture, Jinghong City, Yinchang Mountain, 21°59'6.48"N, 101°14'4.22"E, 1218 m a.s.l., 18 July 2018, *Kai Zhang & Mingsong Wu 00581* (holotype: IBSC!; isotypes: IBSC!, HITBC!).

**Figure 1.**
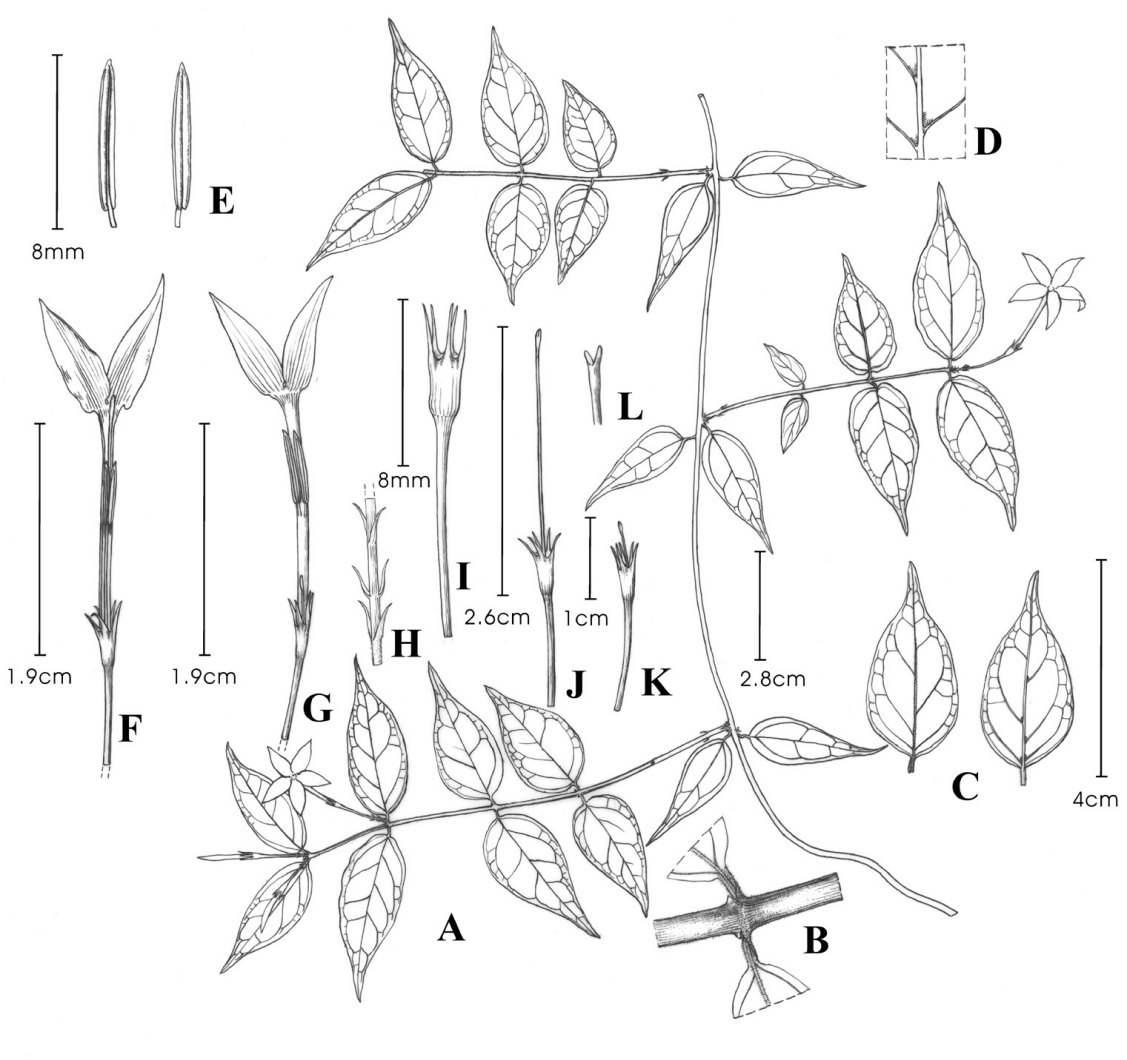
*Jasminum parceflorum*. **A** flowering branch **B** branchlet **C** leaves (adaxial view and abaxial view) **D** vein axils with hair tufts (abaxial view) **E** stamens **F** pin flower **G** thrum flower **H** peduncle with bracts **I** calyx **J** pistil of pin flower **K** pistil of thrum flower **L** stigma. Drawn by Yunxiao Liu.

#### Description.

Shrubs, scandent, evergreen, 1–5 m tall. Branches grayish yellow, terete; branchlets green, slender, pubescent, slightly compressed and inconspicuously grooved when young. Leaves opposite, simple; petiole 1–3 mm long, slightly twisted, articulate near middle, sulcate adaxially, pubescent; leaf blade ovate or lanceolate, sometimes broadly ovate, 2.8–6.3 cm long, 0.9–2.5 cm wide, papery, with entire margin, slightly revolute, base broadly cuneate, rounded or subtruncate, sometimes cuneate or subcordate, slightly oblique, apex acuminate to caudate-acuminate, adaxially pubescent along midvein, and sparsely pubescent near apex when young, abaxially yellow glandular dotted and vein axils with hair tufts; midvein slightly impressed adaxially, elevated abaxially, lateral veins 3–6 pairs, slightly elevated on both surfaces, arcuate-ascendant, anastomosing near margin. Flowers 1 (or 3) in terminal cymes, solitary in leaf axils, dimorphic. Peduncle 1–9 mm long, slightly pubescent. Bracts subulate-filiform, 2–3 mm long, glabrous. Pedicel 1.1–1.6 cm long, glabrous. Calyx campanulate, glabrous; tube 3–4 mm long; lobes 5, linear, (2–) 2.5–4 (–4.5) mm long. Corolla white, salverform; tube 1.3–2.3 cm long, ca. 1.5 mm in diam; lobes 5, lanceolate, 8–11 mm long, 2–3 mm wide, apex argute. Thrum flowers: stamens 2; filaments ca. 1 mm long, glabrous; anthers 12.7–13.6 mm above base of corolla tube, 6.1–7 mm long; stigma 1.5–2.4 mm long, 2-lobed; style filiform, 3.9–5 mm long, glabrous; ovary 0.9–1 mm long, glabrous. Pin flowers: stamens 2; filaments ca. 1 mm long, glabrous; anthers 10.8–11.7 mm above base of corolla tube, 5.4–5.6 mm long; stigma 1.9–2.9 mm long, 2-lobed; style filiform, 13.4–19 mm long, glabrous; ovary 0.9–1 mm long, glabrous. Fruits globose or subglobose, 8–9 mm in diam.

**Figure 2.**
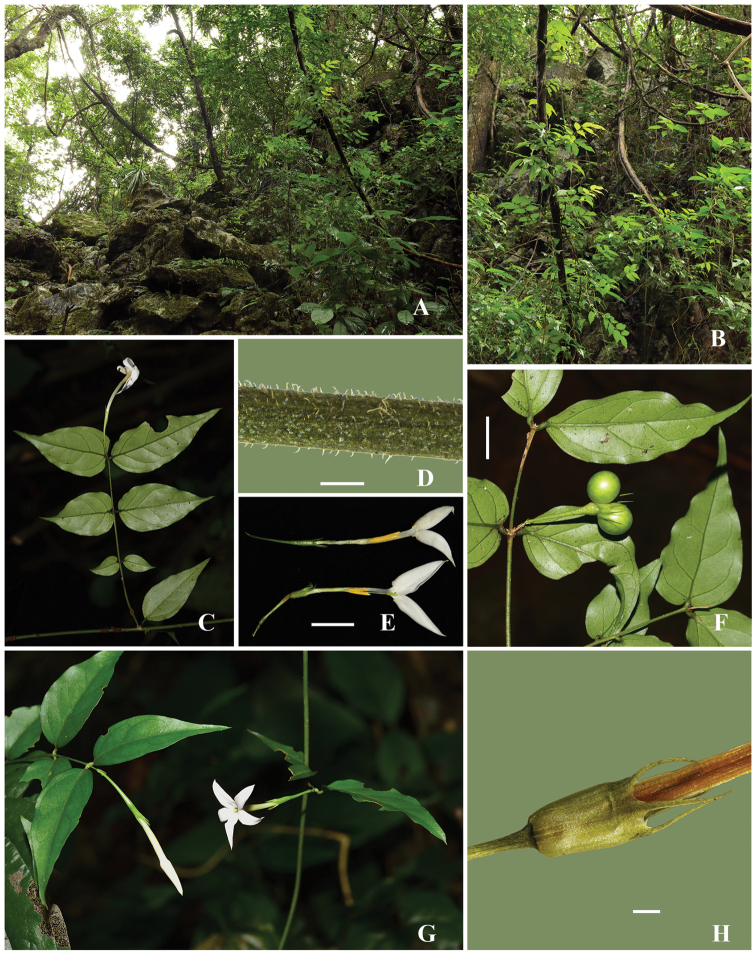
*Jasminum parceflorum*. **A** habitat **B** habit **C** branch with a thrum flower **D** branchlet **E** dissected corolla tubes, thrum flower (upper) and pin flower (lower) **F** fruiting branch **G** branches with pin flowers **H** calyx. Scale bars: 0.5mm (**D**), 1 cm (**E, F**), 1 mm (**H**). Photos by Kai Zhang.

#### Phenology.

Flowering from July to August, fruiting from September to November.

#### Distribution and habitat.

**Figure 3.**
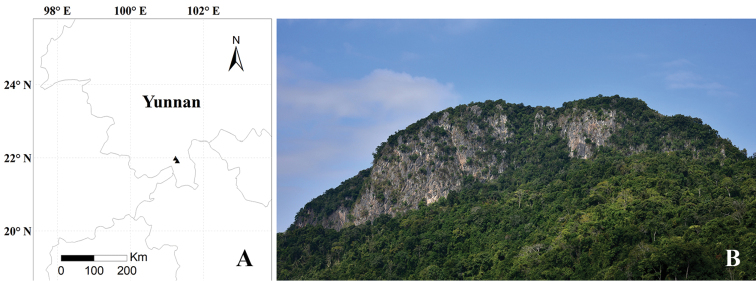
**A** Distribution of *Jasminum parceflorum* in Yunnan Province, China **B** view of Yinchang Mountain.

*Jasminum parceflorum* is currently found in Mengla County and Jinghong City, Xishuangbanna Prefecture, Yunnan Province, China (Fig. 3). It grows in tropical evergreen forests on slopes of limestone mountains, never occurring in open areas such as mountain ridges and forest edges.

#### Conservation status.

Only two populations of the new species were found in Xishuangbanna Prefecture, Yunnan Province, although it may be distributed in adjacent limestone areas. Each population has no more than 200 mature individuals which only occur in limestone forests. According to the IUCN Red List Categories and Criteria (IUCN 2019), *Jasminum parceflorum* is assigned a status of Vulnerable (VU D1).

#### Etymology.

The specific epithet is derived from the fact that its mature individual has fewer flowers in comparison with most other jasmine species.

#### Additional specimens examined.

CHINA. Yunnan Province: Xishuangbanna Prefecture, Jinghong City, Yinchang Mountain, 15 July 2018, *Kai Zhang & Mingsong Wu 00532, 00533* (IBSC!); same locality, 18 July 2018, *Kai Zhang & Mingsong Wu 00582* (IBSC!); same locality, 12 July 2019, *Kai Zhang & Lianxuan Zhou 01177, 01178* (IBSC!); same locality, 13 November 2019, *Kai Zhang 01216* (IBSC!). Mengla County, Lvshilin, 21°54'36.80"N, 101°17'3.83"E, 614 m a.s.l., 13 November 2019, *Kai Zhang 01215* (IBSC!).

## Discussion

Some reproductive characters, such as shape of bracts and calyx lobes, inflorescence type and position are the key taxonomic characters in distinguishing infrageneric taxa of *Jasminum*. The basic inflorescence type of *Jasminum* species is a cyme, which could form a panicle, raceme, corymb, umbel, or head. Most jasmine species have both axillary and terminal cymes, while some species only have terminal cymes, e.g., *J. sambac* (L.) Ait., some species usually only have axillary solitary flowers, e.g., *J. nudiflorum* Lindl., and other species have both axillary solitary flowers and terminal cymes, e.g., *J. rehderianum* Kobuski, although the terminal cymes usually reduce into solitary flowers in this new species. By having terminal 1 (or 3)-flowered cymes and axillary solitary flowers, *J. parceflorum* can be easily distinguished from most jasmine species. It seems to be similar to *J. rarum* in leaf aspect, which was treated as synonym of *J. pierreanum* (Green 2000). Since *J. rarum* differs from *J. pierreanum* in the shape of the leaf base (cuneate to rounded vs. more or less subcordate), and is currently known only from the type collected from Baw Re, Kanchanaburi, Thailand, further studies, particularly observations on living individuals and more specimens of *J. rarum* from the type locality, are needed to reconsider its taxonomic status. Here *J. pierreanum* and *J. rarum* were considered as putative closest allies, and a detailed morphological comparison between them is given in Table 1.

**Table 1.** Comparison of morphological characters among *Jasminum parceflorum*, *J. rarum* and *J. pierreanum*.

**Character**	***J. parceflorum***	***J. rarum***	***J. pierreanum***
Leaf blade base	usually broadly cuneate, rounded or subtruncate, sometimes cuneate or subcordate	broadly cuneate, rounded, sometimes cuneate	more or less subcordate
Calyx lobe	linear, (2–) 2.5–4 (–4.5) mm long	subulate, to somewhat triangular, 0.75 mm long	subulate, to somewhat triangular, 0.25–1 mm long
Dry calyx	ridges absent	ridges present	ridges present
Corolla lobe number	5	6	5 or 6
Inflorescence	terminal 1 (or 3)-flowered cymes and axillary solitary flowers	cymes terminal or axillary, 1–5-flowered	cymes terminal or axillary, (1–) 3–5- or more-flowered

## Supplementary Material

XML Treatment for Jasminum parceflorum
